# Fifteen Minutes of Chair-Based Yoga Postures or Guided Meditation Performed in the Office Can Elicit a Relaxation Response

**DOI:** 10.1155/2012/501986

**Published:** 2012-01-16

**Authors:** Geoffrey W. Melville, Dennis Chang, Ben Colagiuri, Paul W. Marshall, Birinder S. Cheema

**Affiliations:** ^1^School of Biomedical and Health Sciences, University of Western Sydney, Locked Bag 1797, Penrith, NSW 1797, Australia; ^2^Centre for Complementary Medicine Research, University of Western Sydney, Locked Bag 1797, Penrith, NSW 1797, Australia; ^3^School of Psychology, University of New South Wales, Sydney, NSW 2052, Australia

## Abstract

This study compared acute (15 min) yoga posture and guided meditation practice, performed seated in a typical office workspace, on physiological and psychological markers of stress. Twenty participants (39.6 ± 9.5 yr) completed three conditions: yoga, meditation, and control (i.e., usual work) separated by ≥24 hrs. Yoga and meditation significantly reduced perceived stress versus control, and this effect was maintained postintervention. Yoga increased heart rate while meditation reduced heart rate versus control (*P* < 0.05). Respiration rate was reduced during yoga and meditation versus control (*P* < 0.05). Domains of heart rate variability (e.g., SDNN and Total Power) were significantly reduced during control versus yoga and meditation. Systolic and diastolic blood pressure were reduced secondary to meditation versus control only (*P* < 0.05). Physiological adaptations generally regressed toward baseline postintervention. In conclusion, yoga postures or meditation performed in the office can acutely improve several physiological and psychological markers of stress. These effects may be at least partially mediated by reduced respiration rate.

## 1. Introduction

Psychological stress in the workplace is an independent risk factor for cardiometabolic diseases and associated mortality [1–4]. For example, a recent meta-analysis concluded that chronic and excessive work stress can increase the risk of myocardial infarction by 50% [2]. 

The link between stress and disease is mediated by endocrine pathways of the sympathetic nervous system (SNS), including the hypothalamus-pituitary-adrenal (HPA) axis [5, 6]. Cortisol, the main effector of the HPA axis, increases circulating fatty acid and glucose concentrations and inhibits the action of insulin. SNS activation becomes particularly problematic in a sedentary work environment as psychological stress compounded by physical inactivity triggers hyperlipidemia [7] and hyperglycemia, known antecedents to advanced cardiovascular and metabolic diseases [8, 9]. 

Yoga and meditation are forms of complementary and alternative medicine that have become popular in recent decades as methods of managing stress and improving health status. Investigations conducted in nonwork settings have shown that a single session of yoga postures or guided meditation can improve psychological and physiological markers of SNS activation. Acute yoga and/or meditation practice have been shown to reduce perceived stress [10, 11], blood pressure [11], heart rate [11] and respiration rate [12], and increase vagal autonomic modulation evaluated *via* heart rate variability (HRV) [13]. By contrast, sedentary office work has been shown to increase perceived stress, blood pressure, and heart rate and reduce measures of HRV [14, 15]. 

It is often difficult for individuals to commit to regular stress-reducing practice due to time restrictions incurred by work and family life. Brief bouts of yoga or meditation in the workplace could be a potential method to counteract stress. However, to date, no trial has evaluated the effectiveness of such practices.

The purpose of this study was to compare the effect of acute yoga posture and guided meditation practice, performed while seated in the office workspace, on physiological and psychological markers of stress. We hypothesized that both practices would elicit a relaxation response versus the continuation of usual office work (control) evaluated *via* self-perceived stress, blood pressure, heart rate, respiration rate, and time and frequency domains of HRV.

## 2. Methods

### 2.1. Study Design

This was an exploratory study involving a within-subjects crossover design. Participants completed three conditions, including yoga postures, guided meditation, and control, separated by ≥24 hours. Participants were randomized *via* randomization schedule (http://www.randomization.com/) and were blind to their allocation until after the completion of the baseline assessment. The University of Western Sydney Human Research Ethics Committee approved all study procedures, and written informed consent was obtained from all participants. 

### 2.2. Participants

**Inclusion Criteria**

(i) adult (≥18 y), (ii) employed full-time in a sedentary (i.e., office-based) position, (iii) English language sufficient to understand research procedures and provide written informed consent, and (iv) willingness to undergo study protocols.

**Exclusion Criteria**

(i) uncontrolled illness, (ii) use of medication known to alter heart rate or blood pressure, and (iii) acute or chronic medical condition that would impede the assessment of outcome measures.

### 2.3. Procedure

**Figure 1:**
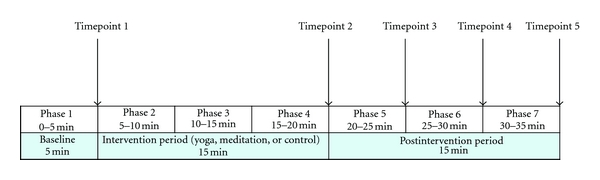
Timeline for the assessment of outcome measures. Noncontinuous measures collected at Timepoints 1 to 5 and continuous measures collected in phases 1 to 7.

A demonstration of the yoga and meditation practices was provided at least one day prior to the first testing session. Each testing session was completed in the office or cubicle workspace of the participant, at a pre-arranged time, during regular office hours. Participants were advised to follow the same meal and beverage patterns on each testing day. All three assessments were completed at precisely the same time of day. Participants were asked to turn off their office and mobile telephones during each testing session. The testing equipment was portable and set-up with minimal disruption to the usual work activities of the participant. A timeline of the study procedure is presented in Figure 1.

#### 2.3.1. Baseline (5 Min)

The purpose of the 5-min baseline assessment was to collect physiological and psychological data reflective of usual office work. Participants were therefore instructed to continue with their work as usual. Minor movements such as mouse clicking or typing were allowed; however, major arm, leg, or trunk movements or rising from the chair, were to be avoided.

#### 2.3.2. Intervention (15 Min)

Immediately following the baseline assessment, participants performed one of the three conditions (yoga, meditation or control) for a 15 min period.

#### 2.3.3. Postintervention (15 Min)

The purpose of the 15 min postintervention assessment was to evaluate the effect of intervention withdrawal. During this period, participants were instructed to resume their usual work adhering to the same movement restrictions applied during the baseline assessment.

### 2.4. Interventions

#### 2.4.1. Yoga

Participants were instructed to perform fifteen minutes of gentle yoga postures while seated in their chair. The intervention, described in detail by Diab [16], involved several postures (e.g., side bend, forward bend, back bend, arms above head and extended); each posture was held for six full breaths (inhalation plus exhalation), approximately from 30 to 60 seconds, and the cycle was repeated as required. A brief relaxation period involving eyes closed and no movement was also included during this intervention. The intervention integrated deep breathing and was specifically designed to induce relaxation [16]. 

#### 2.4.2. Meditation

Participants performed fifteen minutes of guided meditation instructed by a prerecorded mp3 file (Debra McCormack, Sydney, Australia, 2010), delivered via Ipod (Apple, Cupertino, California, USA) and headphones (TDK NC-150; Tokyo Denki Kagaku, Nihonbashi, Japan). The practice emphasized deep breathing, visualization, and alleviating internal dialogue. Participants were instructed to follow the instructions as closely as possible. 

#### 2.4.3. Control

Participants were instructed to continue with their office work with the same movement and talking restrictions as applied during the baseline recording.

### 2.5. Outcome Measures

Perceived stress and blood pressure were collected at timepoints 1 to 5 (T1–T5) (Figure 1). Perceived stress was evaluated by means of a 100 mm visual analogue on which participants rated their state of stress/relaxation at that particular moment, ranging from “Extreme Relaxation” (0 mm) to “Extreme Stress” (100 mm). A score of 50 mm was designated “Neutral”. Systolic and diastolic blood pressures were evaluated *via* auscultation.

Heart rate, respiration rate, and time and frequency domains of HRV were recorded continuously. Data were analysed in seven 5 min phases including 1 × 5 min recording at baseline (P1), 3 × 5 min recordings during the intervention (P2–P4), and 3 × 5 min recordings postintervention (P5–P7).

Respiration rate was evaluated using a PowerLab 26T Advanced Teaching System and a Piezo Respiratory Belt Transducer attachment (ML856 and MLT1132, ADInstruments, Castle Hill, Australia) placed level with the umbilicus. Data were recorded in LabChart 7.1. Respiration rate (inhalation plus expiration) in breaths per minute was calculated for each 5 min phase.

Heart rate and HRV were recorded using a telemetric Polar Heart Rate Monitor (Polar, RS800sd, Kempele, Finland). The polar RS800 is functionally equivalent to the S810 model, which has been validated for short-term recordings of HRV [17, 18]. Pertinent time and frequency domains of HRV were assessed, including SDNN (i.e., standard deviation of normal-normal intervals), Total Power (TP; i.e., the variance of all normal-normal intervals), the Low Frequency (LF) power component (0.04 to 0.15 Hz) measured in absolute (ms^2^) and relative (%) units, the High Frequency (HF) power component (0.15 to 0.4 Hz) measured in absolute (ms^2^) and relative (%) units, and the Low Frequency to High Frequency Ratio (LF : HF). Low measures of HRV including low TP and low SDNN are significant and independent risk factors for coronary artery disease [19] and all-cause mortality [20]. Data were imported into ProTrainer 5 software (Version 5.35.165, Polar, Kempele, Finland) and inspected visually for artefact (caused by ectopic beats, arrhythmic events, electromagnetic radiation, etc.). Data were then imported into Kubios Version 2.0 HRV software (Biosignal Analysis and Medical Imaging Group, Department of Applied Physics, University of Kuopi, Finland) for determination of heart rate, and time and frequency domains of HRV [21].

### 2.6. Statistical Analyses

All available data were included in statistical analyses performed using the Statistical Package for the Social Sciences Version 18.0 (SPSS, Somers, NY). All data were inspected visually and statistically for normality. Normally distributed data were described using mean ± standard deviation or mean ± standard error, as indicated, and nonnormally distributed data were described using median and ranges. Nonnormally distributed continuous variables were log-transformed prior to entry into parametric statistical models. Contrast analysis was used to test the difference in the change score from baseline (T1 or P1) to each subsequent phase or timepoint between the control condition and yoga or meditation separately. Similar contrast analysis was used to compare the difference between yoga and meditation conditions. *P* < 0.05 was considered indicative of statistical significance.

## 3. Results

### 3.1. Participants

Participant characteristics are presented in Table 1. Twenty adults were recruited and completed all procedures. Body mass index (BMI) ranged from 19.8 to 44.1 kg/m^2^ and two individuals fulfilled the criteria for obesity (BMI ≥ 30.0 kg/m^2^). One obese participant also had type 2 diabetes. Additional controlled diseases in the cohort included asthma (*n* = 3), chronic fatigue (*n* = 2), and hypertension (*n* = 1). No participant had a history of tobacco use. The majority of participants held academic positions involving teaching and research and did not engage in yoga and/or meditation practice over the previous year.

### 3.2. Outcome Measures

All data are presented in full in Tables 2 and 3. 

#### 3.2.1. Perceived Stress

**Figure 2:**
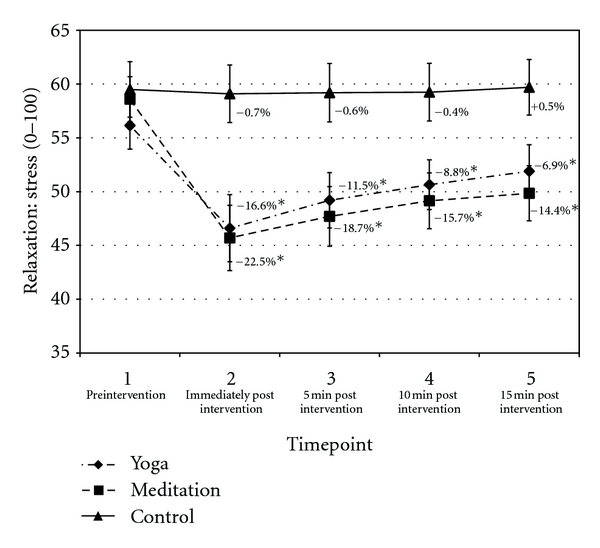
Perceived stress. Data presented as mean ± standard error. Percentage change values refer to change from baseline within condition. *Statistically significant versus control.

Perceived stress was significantly reduced immediately postyoga and postmeditation versus control, and these effects were maintained throughout the postintervention period (Figure 2). There was no significant change in perceived stress between yoga and meditation at any timepoint.

#### 3.2.2. Blood Pressure

Changes in systolic and diastolic blood pressure were not significantly different between yoga versus control or yoga versus meditation at any timepoint; however, both systolic and diastolic blood pressure were significantly reduced in meditation versus control at T3 (see Tables 2 and 3).

#### 3.2.3. Respiration Rate

**Figure 3:**
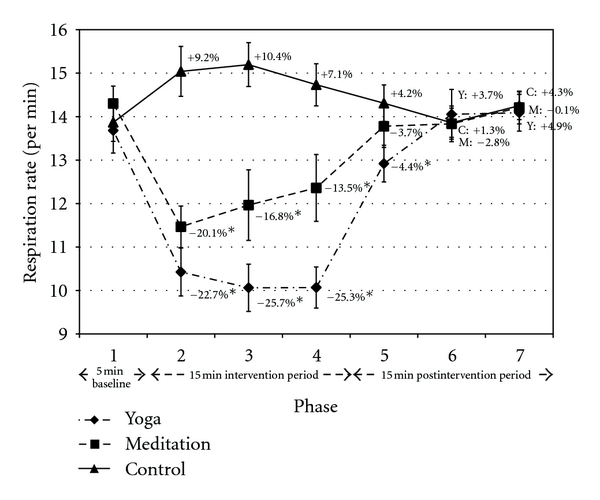
Respiration rate. Data presented as mean ± standard error. Percentage change values refer to change from baseline within condition. *Statistically significant versus control. Y: yoga; M: meditation; C: control.

Respiration rate decreased by 24.6% (mean of P2–P4) during yoga and increased 8.9% during control (Figure 3). Both effects regressed toward the baseline value during the postintervention period. Respiration rate significantly decreased by 16.8% (mean of P2–P4) during meditation versus control (*P* < 0.05) and regressed toward the baseline value postintervention. No significant differences were noted between yoga and meditation.

#### 3.2.4. Heart Rate

**Figure 4:**
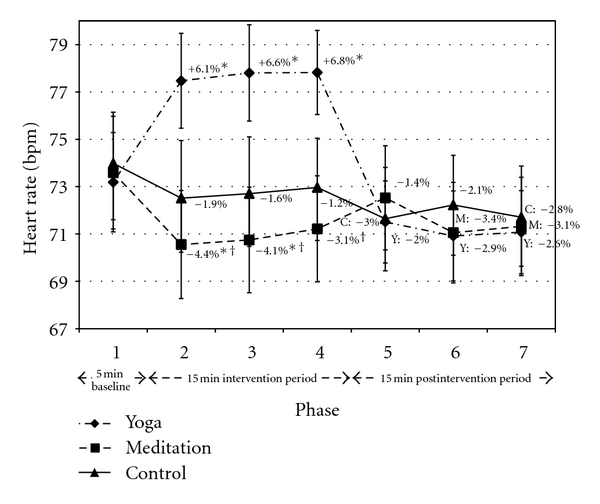
Heart rate. Data presented as mean ± standard error. Percentage change values refer to change from baseline within condition. *Statistically significant versus control. ^†^Statistically significant versus yoga. Y: yoga; M: meditation; C: control.

Yoga significantly increased heart rate by 6.5% (mean of P2–P4) versus control (Figure 4). By contrast, meditation significantly reduced heart rate versus control in P2 and P3, with a trend noted in P4 (−3.9%, mean of P2–P4). Change in heart rate was significantly different between yoga and meditation throughout the intervention period. No condition × time effects were noted postintervention.

#### 3.2.5. HRV Time Domains

**Figure 5:**
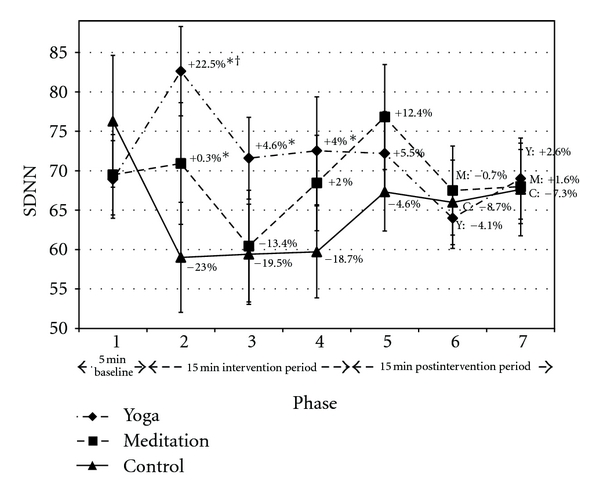
SDNN. Data presented as mean ± standard error. Percentage change values refer to change from baseline within condition. *Statistically significant versus control. ^†^Statistically significant versus meditation. Y: yoga; M: meditation; C: control.

SDNN increased during yoga and decreased during control, and the difference between conditions was significant (Figure 5). The most notable increase in SDNN during yoga (P2, +22.5%) coincided with the initiation of physical postures, while the difference in P3 and P4 was mediated by the large decrease in SDNN during control. The change in SDNN was significantly different between meditation and control during P2 only, and this difference was also mediated by the large decrease in SDNN in control. There was a notable decrease in SDNN during meditation in P3 and trend toward increased SDNN in meditation versus control in P4 and P5. SDNN significantly increased in yoga versus meditation during P2 only, with a trend noted in P3.

#### 3.2.6. HRV Frequency Domains

Total Power (TP) and absolute HF and LF power components were not normally distributed and were therefore normalized *via* logarithmic transformation prior to analysis with parametric statistical models.

**Figure 6:**
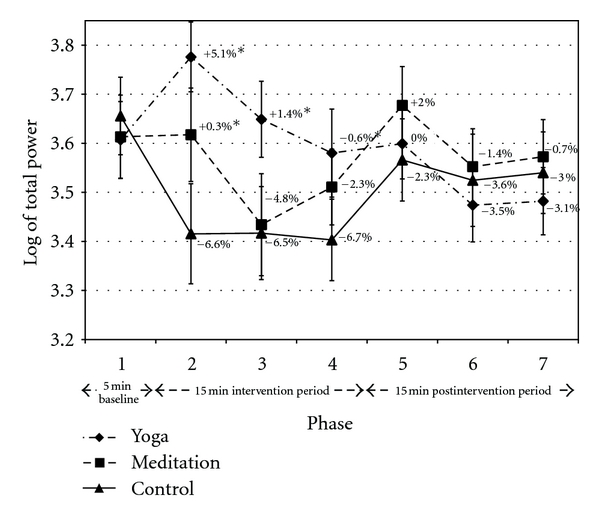
Log-Total Power. Data presented as mean ± standard error. Percentage change values refer to change from baseline within condition. *Statistically significant versus control. ^†^Statistically significant versus meditation. Y: yoga; M: meditation; C: control.

The findings for log-TP were similar to those obtained for SDNN. Log-TP increased during yoga and decreased during control, and the difference was primarily mediated by the large decrease in control (Figure 6). The absolute and relative LF components increased during yoga versus control in P2 and P3 (see Tables 2 and 3). LF measures were not different between yoga and control in P4. Yoga also induced a decrease in the relative HF power component and an increase in LF : HF in P2 and P3. No other effects were noted between yoga and control, except that log-LF and LF : HF were significantly reduced during yoga versus control in P7.

Log-TP was significantly reduced in control versus meditation during P2 only (Figure 6). This change was accompanied by increased or trend toward increased absolute and relative LF components, a trend toward reduced relative HF component, and a trend toward increased LF : HF ratio in meditation versus control (see Tables 2 and 3). No other differences were noted between meditation and control.

Log-TP significantly increased in yoga versus meditation in P2, and this change was accompanied by a trend toward increased absolute and relative LF components and a trend toward reduced relative HF component (see Tables 2 and 3). During P3, log-TP and the absolute and relative LF components significantly increased, while relative HF was significantly reduced, in yoga versus meditation. The LF : HF ratio increased or tended to increase with yoga versus meditation during P2, P3, and P4. No other differences were noted between yoga and meditation.

## 4. Discussion

This study evaluated the effect of brief yoga posture and meditation practice, performed while seated in the office workspace, on physiological and psychological markers of stress. Both yoga and meditation reduced perceived stress versus the control condition (i.e., the continuation of regular office work), and these effects were maintained throughout the 15 min postintervention period (Figure 2). Physiological responses also indicated a relaxation effect during yoga and meditation.

The adaptation of certain physiological outcome measures during yoga or meditation practice, such as SDNN and log-TP, was mediated by increased stress during control. It could, therefore, be hypothesized that the 5 min baseline recording in all conditions reflected an artificial state in which greater parasympathetic modulation may have been the product of interaction between the participant and tester (e.g., touching, for the purpose of evaluating blood pressure). Postintervention responses in every condition demonstrated a return to the baseline value, indicating that interaction between the participant and tester may have exerted relaxation during this period as well. Thus, in a true workplace situation, the parasympathetic influence would likely be lower, as observed in SDNN and log-TP measures during P2–P4.

Both yoga and meditation reduced the respiration rate compared to control (Figure 3). The yoga postures integrated deep breathing, while the guided mediation instructed the listener to slow and deepen their breath. The control condition increased respiration by 8.9%. This may have been accompanied by shallow breathing. Studies have shown that deep breathing techniques can acutely improve baroreceptor sensitivity and reduce blood pressure [22, 23]. By contrast, shallow breathing can increase SNS activity [24]. Studies are required to investigate the differential effects of yoga and meditation versus general deep breathing exercises to determine if the relaxation effects are primarily explained by respiration.

Respiration was not significantly different between yoga postures and meditation at any timepoint (Figure 4). However, respiration gradually increased throughout the meditation intervention while this effect was not observed during yoga. The difference could be attributed to the meditation instruction. Breathing became less emphasized and visualization became more emphasized over the course of the meditation session. Our cohort had minimal experience with meditation, and lack of direct attention on the breath may have contributed to the gradual increase in respiration.

Treatment effects noted for HRV time and frequency domains during yoga and meditation may have been influenced by respiration. Deep breathing is known to increase parasympathetic modulation [25]. Previous studies have attempted to control for respiration [26]. However, this can adversely affect the therapeutic potential of the intervention. SDNN and log-TP both increased in yoga relative to control during the intervention period (Figures 5 and 6 from P2 to P4). This is notable given that reduced ambulatory SDNN and TP are associated with the development of coronary artery disease [19] and all-cause mortality [20].

The improvement of TP during yoga versus control was accompanied by increased or a trend toward increased log-LF, relative LF, and the LF : HF ratio during P2 and P3. This largest increase in these parameters was detected in P2, during the initiation of physical postures. The LF component reflects both sympathetic and parasympathetic modulation [25]. Interventions known to increase sympathetic activity (e.g., vigorous exercise) reduce TP and SDNN and generally increase blood pressure [25]. However, TP and SDNN were increased, while there was no change in blood pressure with yoga, suggesting that changes in the LF component during P2 and P3 of yoga were not primarily due to increased sympathetic inputs. Peng et al. [27] noted increased LF power and reduced respiration rate in experienced meditators during practice. The adaptations were interpreted to reflect increased respiratory sinus arrhythmia and vagal modulation [27]. Similar findings have been documented in a Zen meditation study [28].

The meditation condition significantly increased SDNN and TP versus control during P2 only. These effects were accompanied by an increase in log-LF. The sharp reduction in SDNN and log-TP from P2 to P3 may have been due to the specific instruction, compounded by the relative inexperience of our cohort. From 7:26 to 11:08 min, the guided meditation contained a silent section of no instruction. It may have been difficult for our participants, predominantly inexperienced meditators, to maintain a quiet mind during this period. Thoughts about work or daydreaming may have elicited an increase in SNS activation.

Trends toward increased SDNN and log-TP in meditation versus control were noted in P4 and during the first postintervention phase (P5). Thus, significant improvements during the intervention and postintervention (residual effect) may have been observed with a larger sample size, more experienced meditators, and/or removal of the silent section of the guided meditation.

The yoga intervention resulted in a greater number of physiological benefits than the guided meditation condition. It could be hypothesized that the performance of yoga postures is required to facilitate a quiet mind, particularly in individuals employed in professions that require significant mental focus, such as academic work. This supports the position that individuals in westernized countries benefit from performing yoga postures prior to meditation [29].

Heart rate throughout the intervention period (from P2 to P4) was different between yoga and meditation practice. However, the relatively small (6.5%) increase in heart rate with yoga indicates that the chair-based yoga postures were only a very mild form of exertion. Other differences between yoga and meditation included enhanced time and frequency domains of HRV in the early stages of intervention period (from P2 to P3) favouring a relaxation response in yoga.

Yoga and meditation had a residual effect on perceived stress as the benefits were maintained throughout the 15 min postintervention period (Figure 3). By contrast, physiological adaptations elicited by all conditions (i.e., yoga, meditation, and control) generally regressed to baseline values during the 15 min postintervention period (from P5 to P7). The main exception was respiration rate, which remained significantly reduced in yoga versus control in P5. More experienced practitioners may have derived more residual benefits from intervention, though further research is required to confirm this hypothesis.

Log-LF and LF : HF were significantly reduced in yoga relative to control in P7, while RMSSD was significantly higher in meditation versus control in P6. It is not clear whether these were actual residual effects or anomalies.

Systolic and diastolic blood pressure significantly decreased in the meditation condition versus control at T3 only, while no change was noted in blood pressure with yoga. However, our cohort was normotensive at baseline, and as such an improvement of blood pressure would not be expected. Future studies inducing a stress response just prior to prescribing a stress-reducing intervention may be able to facilitate a rise in systolic and diastolic blood pressure in normotensive office workers in future trials. Studies conducted in patients with hypertension have shown that meditation can reduce systolic and diastolic blood pressure [30, 31].

In summary, this study suggests that participation in 15 minutes of chair-based yoga postures or guided meditation in the office workspace can acutely improve several physiological and psychological markers of stress. These effects may be at least partially mediated by reduced respiration rate. Long-term utilization of these practices to mitigate stress in the workplace may result in significant health-related benefits, including the reduced risk of cardiometabolic diseases. Randomised controlled trials to investigate such hypotheses in various sedentary occupations are recommended.

## Figures and Tables

**Table 1:** Participant characteristics (*n* = 20).

Age (years)	39.6 ± 9.5
Sex (men : women)	8 : 12
Height (cm)	169 ± 12
Body mass (kg)	70.0 ± 16.3
Body mass index (kg/m^2^)^†^	22.5 (19.8–44.1)

Occupation (*n*)	
Academic staff (lecturer and researcher)	11
Administrative staff	4
Researcher	2
Research assistant	2
Campus facility manager	1

Yoga/meditation practice over previous year (*n*)	
Did not practice	14
Practiced 1 to 3 times per month	3
Practiced 1 to 2 times per week	1
Practiced 3 times per week	2

Data reported according to mean ± standard deviations for normally distributed variables.

^†^Nonnormal distribution: median values and range reported.

**Table 2:** Timepoint data.

	T1	T2	T3	T4	T5
Systolic BP (mmHg)					
Control	116.70 ± 12.38	115.25 ± 11.71	116.30 ± 13.48	114.80 ± 11.99	114.85 ± 12.98
Yoga	111.70 ± 13.42	110.85 ± 13.31	109.35 ± 13.51	109.95 ± 10.40	110.35 ± 12.56
Meditation	114.55 ± 12.17	110.15 ± 13.03	109.05 ± 13.32	113.50 ± 11.52	114.15 ± 12.50
*P* (C versus Y)		0.700	0.378	0.942	0.830
*P* (C versus M)		0.187	0.028*	0.753	0.516
*P* (Y versus M)		0.111	0.166	0.777	0.726

Diastolic BP (mmHg)					
Control	76.55 ± 11.17	76.80 ± 10.84	77.95 ± 11.07	77.00 ± 10.20	78.05 ± 10.07
Yoga	75.40 ± 11.52	76.45 ± 11.38	76.30 ± 10.68	76.25 ± 9.35	76.10 ± 11.18
Meditation	77.55 ± 10.67	75.95 ± 11.26	75.60 ± 10.25	76.45 ± 10.18	77.65 ± 10.61
*P* (C versus Y)		0.616	0.806	0.829	0.677
*P* (C versus M)		0.259	0.041*	0.358	0.459
*P* (Y versus M)		0.119	0.223	0.446	0.785

Perceived stress (0–100)					
Control	59.50 ± 11.57	59.10 ± 11.95	59.20 ± 12.17	59.25 ± 11.99	59.70 ± 11.57
Yoga	56.15 ± 9.80	46.60 ± 13.97	49.20 ± 11.57	50.65 ± 10.36	51.90 ± 11.04
Meditation	58.60 ± 9.36	45.70 ± 13.57	47.70 ± 12.46	49.15 ± 11.61	49.85 ± 11.46
*P* (C versus Y)		0.003*	0.007*	0.007*	0.018*
*P* (C versus M)		0.000*	0.000*	0.002*	0.003*
*P* (Y versus M)		0.154	0.088	0.219	0.189

Data presented in mean ± standard deviation; BP: blood pressure; Y: yoga; C: control; M: meditation.

*Change from baseline (T1) statistically significant between groups.

**Table 3:** Phase data.

	P1	P2	P3	P4	P5	P6	P7
Respiration rate							
Control	13.87 ± 1.95	15.04 ± 2.57	15.20 ± 2.26	14.73 ± 2.17	14.31 ± 1.88	13.86 ± 1.49	14.25 ± 1.42
Yoga	13.68 ± 2.32	10.43 ± 2.46	10.06 ± 2.43	10.07 ± 2.12	12.92 ± 1.89	14.06 ± 2.56	14.09 ± 1.89
Meditation	14.30 ± 1.79	11.47 ± 2.14	11.97 ± 3.63	12.36 ± 3.44	13.78 ± 2.18	13.84 ± 1.83	14.21 ± 1.68
*P* (C versus Y)		0.000*	0.000*	0.000*	0.023*	0.573	0.960
*P* (C versus M)		0.000*	0.000*	0.006*	0.106	0.470	0.349
*P* (Y versus M)		0.591*	0.169	0.075	0.623	0.118	0.350

Heart Rate							
Control	73.98 ± 10.65	72.51 ± 10.20	72.71 ± 9.98	72.97 ± 10.01	71.65 ± 9.83	72.23 ± 9.48	71.71 ± 9.28
Yoga	73.19 ± 9.37	77.47 ± 8.95	77.80 ± 9.09	77.82 ± 7.95	71.51 ± 7.75	70.92 ± 8.50	71.08 ± 7.85
Meditation	73.59 ± 9.66	70.56 ± 10.90	70.75 ± 10.70	71.22 ± 9.30	72.53 ± 9.69	71.06 ± 9.38	71.32 ± 9.64
*P* (C versus Y)		0.000*	0.000*	0.000*	0.315	0.570	0.860
*P* (C versus M)		0.032*	0.015*	0.098	0.135	0.369	0.999
*P* (Y versus M)		0.000*	0.000*	0.000*	0.465	0.744	0.870

SDNN							
Control	76.27 ± 37.35	59.01 ± 31.23	59.41 ± 28.41	59.70 ± 26.06	67.32 ± 22.15	65.99 ± 23.96	67.63 ± 26.25
Yoga	68.89 ± 22.02	82.62 ± 25.35	71.59 ± 23.24	72.53 ± 30.59	72.19 ± 23.71	63.98 ± 17.16	69.00 ± 22.98
Meditation	69.49 ± 22.78	70.92 ± 34.50	60.44 ± 31.68	68.45 ± 27.05	76.82 ± 29.79	67.50 ± 25.26	67.99 ± 21.00
*P* (C versus Y)		0.000*	0.009*	0.007*	0.093	0.396	0.191
*P* (C versus M)		0.019*	0.367	0.104	0.086	0.259	0.179
*P* (Y versus M)		0.033*	0.055	0.332	0.354	0.503	0.638

Log-TP (ms^2^)							
Control	3.66 ± 0.35	3.42 ± 0.46	3.42 ± 0.42	3.40 ± 0.37	3.57 ± 0.38	3.53 ± 0.42	3.54 ± 0.37
Yoga	3.61 ± 0.35	3.78 ± 0.32	3.65 ± 0.35	3.58 ± 0.40	3.60 ± 0.32	3.47 ± 0.33	3.48 ± 0.31
Meditation	3.61 ± 0.38	3.62 ± 0.43	3.44 ± 0.47	3.51 ± 0.35	3.68 ± 0.35	3.55 ± 0.35	3.57 ± 0.34
*P* (C versus Y)		0.001*	0.003*	0.026*	0.264	0.977	0.916
*P* (C versus M)		0.013*	0.510	0.202	0.070	0.317	0.377
*P* (Y versus M)		0.112	0.017	0.369	0.222	0.221	0.313

Log-LF (ms^2^)							
Control	3.00 ± 0.38	2.85 ± 0.51	2.84 ± 0.50	2.90 ± 0.40	3.02 ± 0.37	2.93 ± 0.44	2.99 ± 0.41
Yoga	3.06 ± 0.40	3.47 ± 0.40	3.22 ± 0.42	3.11 ± 0.55	3.09 ± 0.41	2.91 ± 0.43	2.88 ± 0.33
Meditation	2.99 ± 0.34	3.13 ± 0.57	2.88 ± 0.69	2.96 ± 0.51	3.08 ± 0.40	3.02 ± 0.41	2.95 ± 0.42
*P* (C versus Y)		0.001*	0.003*	0.234	0.986	0.270	0.047*
*P* (C versus M)		0.048*	0.714	0.594	0.266	0.287	0.751
*P* (Y versus M)		0.072	0.040*	0.437	0.365	0.064	0.138

LF (n.u.)							
Control	69.79 ± 14.31	66.12 ± 15.44	67.52 ± 15.22	71.39 ± 16.22	68.72 ± 17.48	70.67 ± 15.87	70.44 ± 18.24
Yoga	72.68 ± 12.34	88.85 ± 6.75	80.47 ± 13.76	76.75 ± 21.24	76.40 ± 10.76	71.12 ± 14.36	66.77 ± 12.78
Meditation	70.44 ± 12.83	76.32 ± 19.11	63.53 ± 24.40	70.67 ± 18.96	75.78 ± 13.35	70.28 ± 19.50	66.04 ± 16.65
*P* (C versus Y)		0.001*	0.065	0.697	0.301	0.501	0.145
*P* (C versus M)		0.098	0.478	0.801	0.157	0.819	0.326
*P* (Y versus M)		0.056	0.019*	0.455	0.688	0.781	0.707

Log-HF (ms^2^)							
Control	2.60 ± 0.47	2.53 ± 0.53	2.48 ± 0.50	2.45 ± 0.48	2.61 ± 0.45	2.50 ± 0.49	2.55 ± 0.50
Yoga	2.62 ± 0.51	2.52 ± 0.44	2.56 ± 0.44	2.50 ± 0.54	2.55 ± 0.48	2.47 ± 0.44	2.56 ± 0.37
Meditation	2.58 ± 0.53	2.51 ± 0.55	2.59 ± 0.56	2.51 ± 0.45	2.53 ± 0.50	2.57 ± 0.52	2.61 ± 0.42
*P* (C versus Y)		0.582	0.541	0.789	0.146	0.437	0.749
*P* (C versus M)		0.915	0.129	0.374	0.500	0.357	0.365
*P* (Y versus M)		0.620	0.527	0.605	0.766	0.128	0.171

HF (n.u.)							
Control	30.22 ± 14.31	33.89 ± 15.44	32.49 ± 15.22	28.61 ± 16.22	28.71 ± 15.85	29.34 ± 15.87	29.57 ± 18.24
Yoga	27.32 ± 12.34	11.16 ± 6.75	19.54 ± 13.76	23.26 ± 21.24	23.60 ± 10.76	28.89 ± 14.36	33.24 ± 12.78
Meditation	29.56 ± 12.83	23.68 ± 19.11	36.47 ± 24.40	29.33 ± 18.96	24.22 ± 13.35	29.72 ± 19.50	33.96 ± 16.65
*P* (C versus Y)		0.001*	0.065	0.697	0.589	0.501	0.145
*P* (C versus M)		0.098	0.478	0.801	0.361	0.819	0.326
*P* (Y versus M)		0.056	0.019*	0.455	0.688	0.781	0.707

LF : HF							
Control	3.21 ± 2.53	2.55 ± 1.57	3.04 ± 2.44	3.90 ± 2.91	3.27 ± 2.15	3.34 ± 2.06	3.89 ± 3.06
Yoga	3.61 ± 2.63	13.27 ± 12.38	6.00 ± 3.86	8.10 ± 8.80	4.22 ± 2.43	3.38 ± 2.28	2.81 ± 2.81
Meditation	3.12 ± 2.03	7.57 ± 10.89	3.45 ± 3.42	4.21 ± 4.11	4.62 ± 3.35	4.31 ± 3.88	3.26 ± 3.35
*P* (C versus Y)		0.003*	0.015*	0.080	0.380	0.529	0.024*
*P* (C versus M)		0.075	0.593	0.734	0.089	0.254	0.466
*P* (Y versus M)		0.069	0.024*	0.108	0.292	0.107	0.178

Data presented in mean ± standard deviation; Y: yoga; C: control; M: meditation.

*Change from baseline (P1) statistically significant between groups.

## References

[1] Chandola T, Brunner E, Marmot M (2006). Chronic stress at work and the metabolic syndrome: prospective study. *British Medical Journal*.

[2] Kivimäki M, Virtanen M, Elovainio M, Kouvonen A, Väänänen A, Vahtera J (2006). Work stress in the etiology of coronary heart disease—a meta-analysis. *Scandinavian Journal of Work, Environment and Health*.

[3] Rosengren A, Hawken S, Ôunpuu S (2004). Association of psychosocial risk factors with risk of acute myocardial infarction in 11 119 cases and 13 648 controls from 52 countries (the INTERHEART study): case-control study. *The Lancet*.

[4] Kivimäki M, Leino-Arjas P, Luukkonen R, Riihimäki H, Vahtera J, Kirjonen J (2002). Work stress and risk of cardiovascular mortality: prospective cohort study of industrial employees. *British Medical Journal*.

[5] Cohen S, Janicki-Deverts D, Miller GE (2007). Psychological stress and disease. *Journal of the American Medical Association*.

[6] Rosmond R, Björntorp P (2000). The hypothalamic-pituitary-adrenal axis activity as a predictor of cardiovascular disease, type 2 diabetes and stroke. *Journal of Internal Medicine*.

[7] Siegrist J, Peter R, Cremer P, Seidel D (1997). Chronic work stress is associated with atherogenic lipids and elevated fibrinogen in middle-aged men. *Journal of Internal Medicine*.

[8] McEwen BS (1998). Protective and damaging effects of stress mediators. *The New England Journal of Medicine*.

[9] Vrijkotte TGM, van Doornen LJP, De Geus EJC (1999). Work stress and metabolic and hemostatic risk factors. *Psychosomatic Medicine*.

[10] West J, Otte C, Geher K, Johnson J, Mohr DC (2004). Effects of Hatha Yoga and African dance on perceived stress, affect, and salivary cortisol. *Annals of Behavioral Medicine*.

[11] Rizzolo D, Zipp G, Stiskal D, Simpkins S (2009). Stress management strategies for students: the immediate effects of Yoga, humor, and reading on stress. *Journal of College Teaching and Learning*.

[12] Arambula P, Peper E, Kawakami M, Gibney KH (2001). The physiological correlates of Kundalini Yoga meditation: a study of a yoga master. *Applied Psychophysiology Biofeedback*.

[13] Khattab K, Khattab AA, Ortak J, Richardt G, Bonnemeier H (2007). Iyengar yoga increases cardiac parasympathetic nervous modulation among healthy yoga practitioners. *Evidence-Based Complementary and Alternative Medicine*.

[14] Vrijkotte TGM, van Doornen LJP, De Geus EJC (2000). Effects of work stress on ambulatory blood pressure, heart rate, and heart rate variability. *Hypertension*.

[15] Collins SM, Karasek RA, Costas K (2005). Job strain and autonomic indices of cardiovascular disease risk. *American Journal of Industrial Medicine*.

[16] Diab CE (2003). *Three Key Points to Relaxation*.

[17] Gamelin FX, Berthoin S, Bosquet L (2006). Validity of the polar S810 Heart rate monitor to measure R-R intervals at rest. *Medicine and Science in Sports and Exercise*.

[18] Nunan D, Gay D, Jakovljevic DG, Hodges LD, Sandercock GRH, Brodie DA (2009). Validity and reliability of short-term heart-rate variability from the Polar S810. *Medicine and Science in Sports and Exercise*.

[19] Huikuri HV, Jokinen V, Syvänne M (1999). Heart rate variability and progression of coronary atherosclerosis. *Arteriosclerosis, Thrombosis, and Vascular Biology*.

[20] Tsuji H, Venditti FJ, Manders ES (1994). Reduced heart rate variability and mortality risk in an elderly cohort: the Framingham heart study. *Circulation*.

[21] Tarvainen M, Niskanen J (2008). *Kubois HRV Version 2.0 User’s Guide*.

[22] Bernardi L, Porta C, Spicuzza L (2002). Slow breathing increases arterial baroreflex sensitivity in patients with chronic heart failure. *Circulation*.

[23] Schein MH, Gavish B, Herz M (2001). Treating hypertension with a device that slows and regularises breathing: a randomised, double-blind controlled study. *Journal of Human Hypertension*.

[24] Abelson JL, Khan S, Giardino N (2010). HPA axis, respiration and the airways in stress—a review in search of intersections. *Biological Psychology*.

[25] Malik M, Camm AJ, Bigger JT (1996). Heart rate variability. Standards of measurement, physiological interpretation, and clinical use. *European Heart Journal*.

[26] Sakakibara M, Takeuchi S, Hayano J (1994). Effect of relaxation training on cardiac parasympathetic tone. *Psychophysiology*.

[27] Peng CK, Henry IC, Mietus JE (2004). Heart rate dynamics during three forms of meditation. *International Journal of Cardiology*.

[28] Cysarz D, Büssing A (2005). Cardiorespiratory synchronization during Zen meditation. *European Journal of Applied Physiology*.

[29] Borg-Oliver S, Machliss B (2005). *Applied Anatomy & Physiology of Yoga*.

[30] Schneider RH, Alexander CN, Staggers F (2005). A randomized controlled trial of stress reduction in African Americans treated for hypertension for over one year. *American Journal of Hypertension*.

[31] Schneider RH, Staggers F, Alexander CN (1995). A randomized controlled trial of stress reduction for hypertension in older African Americans. *Hypertension*.

